# ﻿An attempt of DNA barcodes based geographical origin authentication of the Chinese caterpillar fungus, *Ophiocordycepssinensis*

**DOI:** 10.3897/imafungus.16.144783

**Published:** 2025-03-31

**Authors:** Yi Li, Jiao-Jiao Lu, Ya-Bin An, Lan Jiang, Hai-Jun Wu, Ke Wang, Dorji Phurbu, Jinmei Luobu, Chao Ma, Rui-Heng Yang, Cai-Hong Dong, Yi-Jian Yao

**Affiliations:** 1 School of Food Science and Engineering, Yangzhou University, Yangzhou, Jiangsu, China Institute of Microbiology, Chinese Academy of Sciences Beijing China; 2 State Key Laboratory of Mycology, Institute of Microbiology, Chinese Academy of Sciences, Beijing, China Yangzhou University Yangzhou China; 3 Nagqu Inspection and Testing Center, Nagqu, Xizang, China Nagqu Inspection and Testing Center Nagqu China; 4 Qinghai Province Ecological and Environmental Monitoring Center, Xining, Qinghai, China Qinghai Province Ecological and Environmental Monitoring Center Xining China; 5 Tibet Plateau Institute of Biology, Lhasa, Xizang, China Tibet Plateau Institute of Biology Lhasa China

**Keywords:** Barcoding, biodiversity, COI, ITS, Tibetan Plateau

## Abstract

*Ophiocordycepssinensis* is one of the best-known traditional Chinese medicines with distribution confined to the Tibetan Plateau and its surrounding regions. Harvesting the fungus contributes greatly to the livelihood of local communities. The quality and price varies amongst different production regions, usually resulting in an intentional mix-up of its production locality during trading processes, which leads to a demand of developing a reliable way that can trace the geographical origin of this fungus. In the present study, a DNA barcoding-based method applying two universal DNA barcodes for identifying fungal and insect, respectively i.e. the nuclear ribosomal internal transcribed spacer (ITS) and the mitochondrial cytochrome oxidase I (COI), was evaluated and used for geographical origin authentication of *O.sinensis*. A total of 24 ITS and 78 COI haplotypes were recognised from 215 individuals collected from 75 different geographic localities (county level). Ninety-nine haplotypes were defined using the combination of ITS and COI, discriminating the 75 investigated production counties into 99 distinct regions. A “core” production region was recognised which covers areas of Nagqu and Qamdo in Xizang, Yushu and Guoluo in Qinghai, Gannan (Maqu and Xiahe) in Gansu and certain regions in Nyingch (Bomi and Zayü) and Lhasa (Damxung) in Xizang and Garzê (Sêrxü) in Sichuan Province. Haplotype analyses using the combined barcodes of ITS and COI showed an excellent performance in the geographical origin authentication of *O.sinensis* and the definition of “core” and “non-core” production region.

## ﻿Introduction

*Ophiocordycepssinensis* (Berk.) G.H. Sung, J.M. Sung, Hywel-Jones & Spatafora [≡ *Cordycepssinensis* (Berk.) Sacc.] (Sung et al. 2007) is a fungus that parasitises underground dwelling larvae of moths (Lepidoptera), especially *Thitarodes* species (Wang and Yao 2011). The fungus has been traditionally used as a tonic for almost 2000 years (Pegler et al. 1994), commonly for the treatment of kidney and lung problems (Committee of Pharmacopeia, Chinese Ministry of Health 1964, 2005). Recent studies have shown that it possesses various pharmacological effects, including antioxidant (Dong and Yao 2008), anti-aging (Jian et al. 2018), anti-tumour (Wu et al. 2007; Ji et al. 2021), immunomodulating (Kuo et al. 1996; Kuo et al. 2007; Wu et al. 2014; Chen et al. 2022), hypoglycaemic (Zhang et al. 2006), hypotensive and vasorelaxant activities (Chiou et al. 2000).

Due to the increasing demand and the significant decrease in wild resources, the price of natural *O.sinensis* products has increased sharply during the past three decades. It has become the most precious fungus worldwide and the top-rated products are even sold at a price higher than gold. Collecting and trading the fungus has become one of the most important income sources for local communities in production areas (Winkler 2008; Cannon et al. 2009; Weckerle et al. 2010; Bohra et al. 2014; Shrestha and Bawa 2014). The species also plays an indispensable role in maintaining the stability of the ecosystem of the Tibetan Plateau (Li et al. 2021b). However, the recent global climate change and over-harvesting have resulted in an apparent habitat loss of the species (Yan et al. 2017; Hopping et al. 2018). The fungus has, thus, been assessed as vulnerable (UV) by the International Union for Conservation of Nature and Natural Resources (Yang 2020), the national Red List of macrofungi in China (Yao et al. 2020) and the Red List of biodiversity of Yunnan Province of China (https://www.cas.cn/yx/201705/t20170522_4602412.shtml). The fungus has also been listed as endangered under the second class of state protection by Chinese government since 1999 (State Forestry Administration and Ministry of Agriculture 1999). Different management strategies have been developed for better utilisation and conservation of the natural resource of this fungus, both in China and in other Himalayan countries (Childs and Choedup 2014; Li et al. 2021b).

Due to the host specificity and the limitations of its living conditions, *O.sinensis* is confined to the alpine shrub and alpine meadow areas on the Tibetan Plateau and its surrounding regions, usually found at altitudes from above 3,000 m to the snow line (Li et al. 2011). The species was reported from Qinghai, Xizang, Sichuan, Gansu and Yunnan in China and the Himalayan countries of Nepal, Bhutan and India (Li et al. 2011). The price of natural *O.sinensis* varies greatly due to its quality variance. Products with the highest quality produced from Nagqu, Xizang (1100–1300 individuals per kg) is selling at a price of 860,000 RMB (~ US$ 117,800) per kg at present, while low-quality products from the same area (~ 5000 individuals per kg) can be purchased at 165,000 RMB (~ US$ 22,600) per kg at local markets (information were from a middleman who sells caterpillar fungus in Lhasa, Xizang on 30 Sept 2023). The top-rated products (~ 2000 individuals per kg) were even valued up to 998,000 RMB (~ US$ 145,000) per kg in 2017 by Tongrentang Pharmaceutical Co., the most well-known pharmacy shop that sells traditional Chinese medicine (Li et al. 2021b).

Although significant genetic variance was observed within this species (Zhang et al. 2009; Quan et al. 2014a, b; Dai et al. 2020) which would probably result in intrinsic differences, the quality and price of *O.sinensis* is mainly graded according to their extrinsic properties, including size, colour, smell and maturity. Those properties are usually considered as highly relevant to their geographic origin. Specimens from Nagqu in Xizang, Yushu in Qinghai and certain other restricted areas, for example, Gyaca County in Shannan, Xizang and Darlag County in Guoluo, Qinghai, are generally considered to be of higher quality and are much more expensive than those from other areas. Those areas were widely recognised as “core” production regions by traders and the general consuming public. Individuals from the core region are usually larger in size compared with the non-core region and the larval host part is firmer with saturated yellow to yellowish-brown colour. The intrinsic quality of the core region is also higher, exhibiting lower levels of heavy metals, such as lead (Pb), cadmium (Cd) and arsenic (As) (Wang et al. 2014) and higher levels of pharmacologically active substances comparing with individuals from non-core regions (unpublished data). Since specimens of different production regions are hardly being distinguished (if not impossible) from their appearance, it is pretty common that unscrupulous traders would mix individuals from other areas and label the mixture as produced from a deceptive better core area such as “Nagqu” or “Yushu”. Undoubtedly, this practice significantly harmed the customers’ benefits and resulted in a loss of consumer trust. The current applied morphology-based methods to discriminate the fungus’ production locality largely rely on the personal experience accumulated over the years. It is difficult for ordinary consumers and market managers to master and may give unreliable identification, making it practically inapplicable, especially when legal disputes are involved. In these circumstances, an accurate identification method of the production locality is obviously needed to standardise the market and protect the consumers’ benefit. Then, this raises an important issue: whether the core and non-core production regions of *O.sinensis* could be distinguished and whether the specific geographic production localities could be traced.

Up to now, different technologies have been developed for geographical origin authentication for this fungus, including using a near-infrared reflectance (NIR) spectroscopy analysis of the methanol extracts (Wang et al. 2006), a random amplified polymorphic DNA (RAPD) fingerprinting analysis (Qian et al. 2011), a nitrogen and carbon isotope analyses by isotope ratio mass spectrometry (Li et al. 2014), a content ratio analysis of methionine to total amino acids (Shi et al. 2021; Ding et al. 2023), a non-targeted metabolomics from UPLC-QTOF-MS (Wang et al. 2024a) and the mineral element analysis (Wang et al. 2024b). These studies either used limited sampling or were only able to classify the geographical origin to the provincial level. A fine-scaled geographical origin authentication not only calls for a more sensitive method, but also need a better sampling which covers the entire production region.

DNA barcoding is a rapid, accurate and cost-effective species identification method (Hebert et al. 2003). In addition to species identification, it could also provide valuable information for analysing population-level variation in some cases (Hajibabaei et al. 2007; Rach et al. 2008; Craft et al. 2010). DNA barcodes may also contain geographical information since population variances may be driven by geographic isolation and, thus, could be used for geographical origin authentication. The nuclear ribosomal internal transcribed spacer (ITS) and the mitochondrial cytochrome oxidase I (COI) are the two universal DNA barcodes for identifying fungal (Schoch et al. 2012) and insects (Hebert et al. 2003) species, respectively. Earlier studies have shown that the intraspecific ITS variation is relatively small in *O.sinensis* (Kinjo and Zang 2001) or so-called “highly homologous” regardless of geographical origin (Chen et al. 2004), very limited ITS haplotypes have been recognised in these studies. However, significant genetic divergences and more ITS haplotypes have been identified within the species when larger sampling sizes were applied in a number of more recent studies (Zhang et al. 2009; Quan et al. 2014b; Dai et al. 2020). While the ITS sequences are not sufficient for geographical origin authentication to an ideal level, for instance, to the county level, other molecular markers are required to increase the discriminability. The diversity of the host insects was far greater than the fungus itself as revealed by COI and other mitochondrial DNA fragments, such as cytochrome c oxidase subunit II (COII) and cytochrome *b* (Cytb) (Quan et al. 2014a, b; Zhang et al. 2014; Dai et al. 2019). At least fifty-seven species in seven genera of the family *Hepialidae* have been recognised as potential hosts of the fungus, according to a literature review (Wang and Yao 2011). Mitochondrial DNA fragments from the host insects thus would probably give more information on geographical origin authentication than the fungal ITS.

Since DNA from both the fungus and its host insects can be obtained from single individuals, a better resolution would be achieved if the combined ITS and COI data were used rather than using a single barcode. In this study, the ITS and COI sequences were obtained from a total of 215 sample individuals collected from 75 different counties of five production provinces and haplotypes were defined and used for geographical origin authentication of this precious fungus. The results would not only provide valuable information for geographical origin authentication, but also benefit conservation of this species.

## ﻿Materials and methods

### ﻿Sampling

A total of 215 individuals were included in this study representing 75 different geographic localities (county level). These samples were collected from 14, 43, 13, 3 and 2 counties in the provinces of Qinghai (111), Xizang (74), Sichuan (19), Gansu (9) and Yunnan (2) (Suppl. material 1), respectively, during the years 2000 to 2015. Geographical information was recorded in detail and provided in Suppl. material 2. A portion of the specimens was bought from local harvesters; thus, the accurate latitude, longitude and elevation for those samples were missing. All specimens were dried with silica gel (Li et al. 2020) and preserved in our lab.

### ﻿DNA extraction and PCR amplification

Total genomic DNA was extracted from dried specimens using the modified CTAB method (Yao et al. 1999). The universal fungal primer pairs ITS5 and ITS4 (White et al. 1990) were used for internal transcribed spacer (ITS) amplification and the primer pairs BI1834 and TH2928 (Folmer et al. 1994) were used for amplification of COI sequences of the host insects. Amplification was performed using a thermal cycler (Eppendorf) in a 25 μl PCR reaction that contained 12.5 μl 2×Taq PCR Master Mix (Tiangen Biotech Co., Ltd., China), 0.25 μl of each primer (10 μM) and 1 μl diluted DNA template. The PCR condition used for ITS was: 7 min at 94 °C; 30 cycles of 94 °C for 30 s, 55 °C for 45 s and 72 °C for 30 s; and a final extension at 72 °C for 10 min. In addition, the conditions for COI were: 2 min at 94 °C; 30 cycles of 95 °C for 30 s, 45 °C for 45 s and 72 °C for 1 min; and a final extension at 72 °C for 10 min. Purification and a direct Sanger sequencing using both primers (the same as PCR primers) were conducted by Shanghai Biozeron Biotechnology Company (Shanghai, China). Sequences obtained in this study have been deposited in GenBank under accession numbers OR652460–OR652582 (ITS), OR669738–OR669952 (COI) (Suppl. material 2).

### ﻿Haplotype definition and classification

ITS and COI sequences were sequenced in both directions and assembled with SeqMan 6.1 module of the Lasergene (DNA Star Inc. WI, USA) software package. Primer sequences were excluded and ambiguous base pairs (bp) were manually checked and edited, based on sequencing chromatograms in BioEdit 7.0.9.1 (Hall 1999). The software DAMBE v.4.2.13 (Xia and Xie 2001) was used to identify the haplotypes. Only identical sequences with 100% similarity were recognised as the same haplotype. ITS Haplotype numbering followed Li et al. (2021) and COI haplotypes were defined and numbered (sorted) in this study according to the frequencies of occurrence.

### ﻿Phylogenetic analyses

Representative sequences of all identified haplotypes were aligned with ClustalW (Thompson et al. 1994) and the alignment was manually refined within BioEdit. Phylogenetic reconstruction of haplotypes was performed using Maximum Likelihood ML) and Bayesian Inference (BI) for both ITS and COI fragments. ML analysis was conducted with RAxML v. 7.2.6 (Stamatakis 2006) using the GTR + G model to obtain the best tree (Li et al. 2020). Bootstrap support (BS) values were calculated with 1000 re-sampling iterations. BI of phylogenetic relationships was performed using the programme MrBayes v. 3.2.6 (Ronquist and Huelsenbeck 2003) with the same substitution model. Two parallel runs with four chains were carried out for 10,000,000 generations. Each chain was sampled every 1000 generations and the first 20% of the trees were discarded as burn-in. The average standard deviation reached below 0.01 indicating a convergence of the two Bayesian runs. A strict consensus tree with branch lengths and posterior probabilities (PP) was obtained with the sumt command. The three phylogenetically closely-related species, i.e. *Ophiocordycepsemeiensis*, *O.lanpingensis* and *O.laojunshanensis*, were used as outgroup taxa for ITS phylogeny (Li et al. 2020, 2021a). The phylogenetic tree of COI was un-rooted.

### ﻿Abbreviations

**BI** Bayesian Inference

**bp** Base pair

**BS** Bootstrap support

**COI** Cytochrome c oxidase subunit I

**COII** Cytochrome c oxidase subunit II

**CTAB** Cetyl trimethylammonium bromide

**Cytb** Cytochrome *b*

**GTR + G** General time reversible model with gamma distributed substitution rates

**ITS** Internal transcribed spacer

**ML** Maximum Likelihood

**NIR** Near-infrared reflectance

**PCR** Polymerase chain reaction

**PP** Posterior probabilities

**RAPD** Random amplified polymorphic DNA

**UV** Vulnerable

## ﻿Results

### ﻿Haplotype classification and distribution

The lengths for ITS and COI sequences were 580 bp and 1009 bp, respectively, after excluding primers. According to DAMBE analyses, 24 ITS haplotypes (F01–F13, F15–F17, F19–F26) were recognised from 215 *O.sinensis* specimens. The haplotypes F01 and F02 were the two most dominant ITS, represented by 123 (57.2%) and 44 (20.5%) individuals. Other ITS haplotypes were represented by 1–10 individuals (Suppl. material 2). Haplotype F01 was spread over a vast area of 46 counties of four provinces, including Gansu (2), Qinghai (6), Sichuan (8) and Xizang (30); those areas are primarily located in the central part of the whole production region (Suppl. material 2). F02 spread over 11 counties of three provinces of Gansu (1), Qinghai (8) and Sichuan (2); those counties are mostly surroundings of the Qinghai Lake, except for the two counties of Sichuan (Zamtang and Batang) (Suppl. material 2). Amongst the five provincial production areas in China, Xizang was found to have the maximum number (14 out of 24) of ITS haplotypes, followed by Qinghai (8), Sichuan (7), Gansu (2) and Yunnan (2) Provinces (Suppl. material 1).

The host insects showed greater diversity than the parasite fungus, with 78 COI haplotypes being recognised. A total of 20, 41, 15, 4 and 2 COI haplotypes were identified from 111, 74, 19, 9 and 2 individuals collected from Qinghai, Xizang, Sichuan, Gansu and Yunnan, respectively (Suppl. material 1). The four most dominant COI haplotypes, i.e. H01, H02, H03 and H04, were represented by 45, 34, 17 and 14 individuals, respectively. The other haplotypes (H05–H78) were represented by 1–8 individuals (Suppl. material 2). A majority (75.6%) of the COI haplotypes (H20–H78) was represented by a single individual (Suppl. material 2).

Combined haplotypes were also defined using both ITS and COI sequences. A total of ninety-nine combined haplotypes have been identified. The four most abundant combined haplotypes were F01H02, F01H03, F01H04 and F02H01. F01H02 was represented by 33 individuals from four counties (Darlag, Gadê, Maqên and Zadoi) in Guoluo Prefecture in Qinghai, accounting for 15.3% of the total sampling. F01H03 was represented by 16 individuals collected from Guoluo in Qinghai (15) and Xiahe in Gansu (1), accounting for 7.4% of the total individuals. F01H04 was represented by 13 individuals (accounting for 7.0%) collected from 10 counties of Qinghai and Xizang. F02H01 was represented by 40 individuals which were collected from one county of Gansu (Minle) and seven counties of Qinghai (Gangca, Menyuan, Qilian, Huzhu, Gonghe, Tianjun, Datong), accounting for 18.6% of the included samples (Suppl. material 2). Considering that the sampling was imbalanced amongst different counties, for example, a total of 57 individuals were sequenced from Maqên County in Guoluo, Qinghai, whereas as many as 40 counties were sampled with only one individual being sequenced. In other words, the most abundant haplotype (calculated in the number of sequenced individuals) may not be the most widely distributed. The two most commonly distributed haplotypes are F01H04 and F02H01, respectively.

### ﻿Phylogenetic analyses

**Figure 1.**
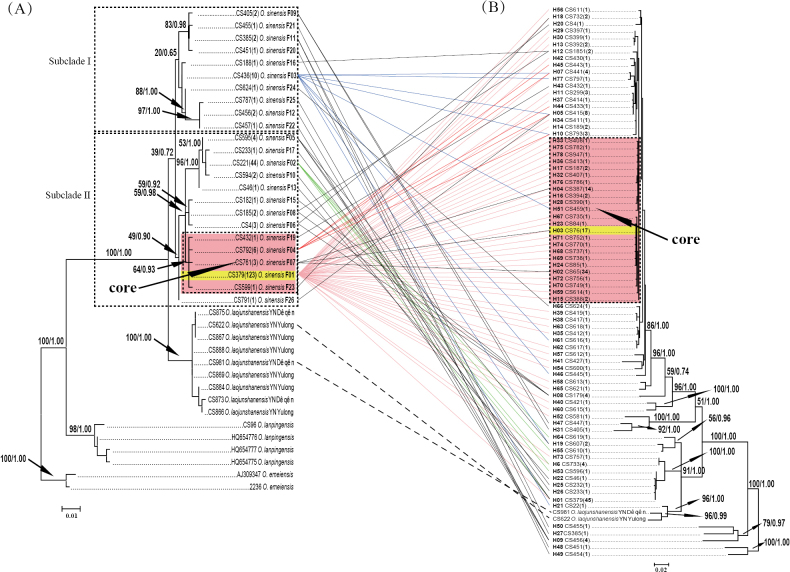
Phylogenetic trees constructed by ITS (**A**) and COI (**B**) haplotypes. ITS and COI sequences from the same individuals are connected with lines. The dashed lines are *Ophiocordycepslaojunshanensis*. The pink, green, blue and red solid lines represent the four most abundant ITS haplotypes F01, F02, F03 and F04, respectively. Numbers above branches are Bayesian posterior probabilities shown as percentages (left) and bootstrap values calculated from the ML analysis of 1000 replicates (right). Haplotypes in a highlighted box are those delimited as representing “core” production regions in this study. The number of specimens of each haplotype sequenced is given in brackets. The yellow highlighted ITS (F01) and COI (H03) haplotypes are identical to the epitype designated by Li et al. (2021).

A representative individual for each haplotype was selected to construct phylogenetic relationships. The BI consensus tree showed a similar topology to the best-scoring ML tree for both fragments (ITS and COI), but had higher supporting values for each clade (Fig. 1). The three outgroup species were all supported as monophyletic with high support values (BS = 98%–100%, PP = 1.00, Fig. 1). Twenty-four haplotypes corresponding to the 215 individuals of *O.sinensis* formed a distinct clade with very low support values (BS = 39%, PP = 0.72, Fig. 1). *Ophiocordycepslaojunshanensis* was displayed as the closest relative of *O.sinensis*. Two major subclades (I and II) were recognised from the ITS phylogenetic tree, but were only weakly supported in ML and Bayesian analyses (Fig. 1).

### ﻿Determination of core and non-core production regions and geographical origin authentication

**Figure 2.**
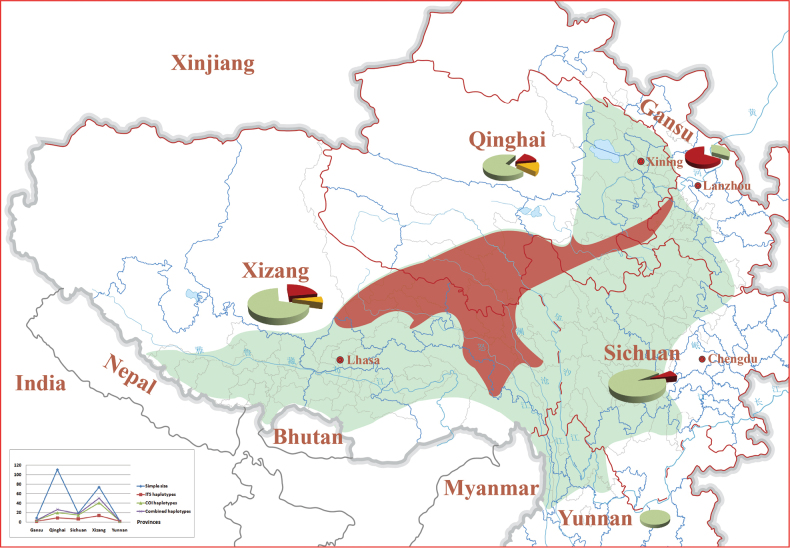
Sampling sizes, haplotype numbers and core/non-core definition for each province. Red shadow indicates the “core” production region, green coloured shadow indicates the “non-core”. Sector graph shows the proportion of the core and non-core region in county level, the yellow colour indicates “mixed” county which contains both the core and non-core production region. The line chart in the lower left corner shows the number of sampling sizes, ITS, COI and the combined haplotypes for each province.

**Figure 3.**
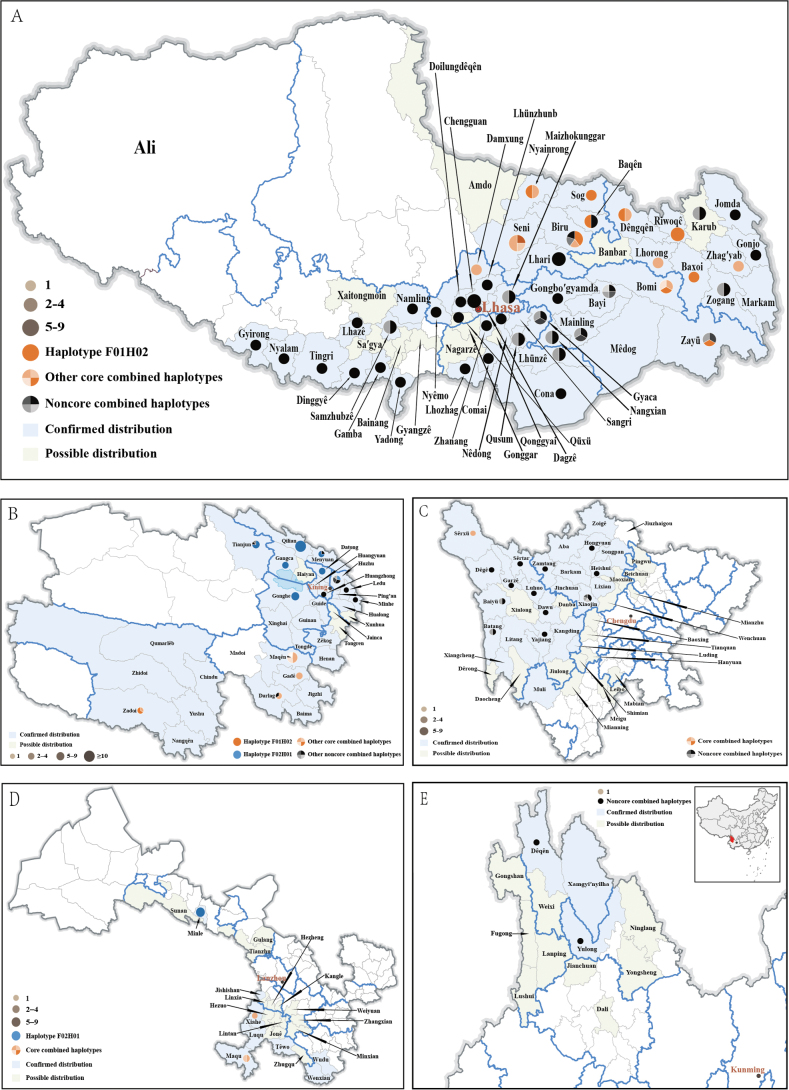
Distribution and haplotype analyses (county level) of *Ophiocordycepssinensis* in Xizang (**A**), Qinghai (**B**), Sichuan (**C**), Gansu (**D**) and Yunnan Provinces (**E**).

The core production refers to those regions that produce the Chinese caterpillar fungus with high qualities. Since the Chinese caterpillar fungus is a product of the parasitism of larvae of ghost moths (*Hepialidae*) by *O.sinensis*, the quality may be associated with the genetic diversity of both the fungus and its host insects. It is quite reasonable to use ITS and COI to determine the core and non-core production regions. In comparison, it is difficult to delimit which haplotypes could represent the “core” in the practical implementation. According to our field experiences with the vast production areas during the past 20 years, it is generally considered that the adjacent regions of Qinghai and Xizang produce fungus of higher quality than other areas. Thus, the main haplotypes identified from those areas were considered hallmarks of the “core” production. Further, the “core” haplotypes should also meet the requirement that they are from the core clades in the ITS and COI phylogenetic trees. According to these criteria, the core ITS haplotypes are F01, F04, F07, F19 and F23 and the core COI haplotype includes H02–H04, H15–H17, H23, H24, H28, H32, H33, H36, H51, H59, H67–H72, H74–H76 and H78 (Fig. 1). The combined haplotypes F01H02, F01H03, F01H04, F01H15, F01H16, F01H17, F01H23, F01H24, F01H28, F01H32, F01H33, F01H36, F01H59, F01H67, F01H68, F01H69, F01H70, F01H71, F01H72, F01H74, F01H75, F01H76, F01H78, F07H02, F07H03 and F07H04 were then defined as the “core” and the core production region would, thus, be confined to areas of Nagqu and Qamdo in Xizang, Yushu and Guoluo in Qinghai, Gannan (Maqu and Xiahe) in Gansu and certain regions in Nyingch (Bomi and Zayü) and Lhasa (Damxung) in Xizang, Garzê (Sêrxü) in Sichuan; other regions were then recognised as “non-core” (Figs 2, 3, Table 1). It is noteworthy that certain counties contained both “core” and “non-core” haplotypes, such as Darlag and Maqên in Guoluo, Baqên and Biru in Nagqu and Zayü in Nyingch (Fig. 3, Table 1).

**Table 1.** Geographical origin authentication of the Chinese caterpillar fungus in county level.

Distribution (county level)	ITS haplotypes	COI haplotypes	Combined haplotypes	Sample size	Classification	Core production determination
Gansu, Gannan, Maqu	F01	H23, H24	F01H23, F01H24	2	Unique	core
Gansu, Gannan, Xiahe	F01	H03	***F01H03*** ^a^	1	Shared	core
Gansu, Zhangye, Minle	F02	H01	***F02H01*** ^a^	6	Shared	non-core
Qinghai, Guoluo, Darlag	F01, F02	H02, H06	***F01H02***^b^, F02H06	3	1 Unique+1 Shared	mixed
Qinghai, Guoluo, Gadê	F01	H02	***F01H02*** ^b^	3	Shared	core
Qinghai, Guoluo, Maqên	F01, F07	H01, H02, H03, H06, H52, H67, H68, H69, H70, H71, H72, H73, H74	F01H01, ***F01H02***^b^, ***F01H03***^a^, F01H06, F01H52, F01H67, F01H68, F01H69, F01H70, F01H71, F01H72, F01H73, F01H74, F07H02, F07H03	57	13 Unique+2 Shared	mixed
Qinghai, Haibei, Gangca	F02	H01	***F02H01*** ^ab^	3	Shared	non-core
Qinghai, Haibei, Menyuan	F02, F13	H01, H22	***F02H01***^ab^, F13H22	4	1 Unique+1 Shared	non-core
Qinghai, Haibei, Qilian	F02	H01	***F02H01*** ^ab^	12	Shared	non-core
Qinghai, Haidong, Huzhu	F02, F05, F10	H01, H53	***F02H01***^ab^, F02H53, F05H01, F10H01	7	3 Unique+1 Shared	non-core
Qinghai, Haidong, Ledu	F23	H18	F23H18	1	Unique	non-core
Qinghai, Haidong, Minhe	F01	H54	F01H54	1	Unique	non-core
Qinghai, Hainan, Gonghe	F02	H01	***F02H01*** ^ab^	5	Shared	non-core
Qinghai, Haixi, Tianjun	F02, F05, F17	H01, H25, H26	***F02H01***^ab^, F05H25, F17H26	8	2 Unique+1 Shared	non-core
Qinghai, Xining, Datong	F02	H01	***F02H01*** ^ab^	3	Shared	non-core
Qinghai, Xining, Huangzhong	F01	H18	F01H18	1	Unique	non-core
Qinghai, Yushu, Zadoi	F01	H02, H04	***F01H02***^b^, ***F01H04***^a^	3	Shared	core
Sichuan, Aba, Heishui	F05	H19	F05H19	1	Unique	non-core
Sichuan, Aba, Hongyuan	F03	H19	F03H19	1	Unique	non-core
Sichuan, Aba, Xiaojin	F06, F08, F15	H08, H12	F06H08, F08H08, F08H12, F15H08	5	Unique	non-core
Sichuan, Aba, Zamtang	F02	H55	F02H55	1	Unique	non-core
Sichuan, Garzê, Baiyü	F01, F03	H61, H62	F01H62, F03H61	2	Unique	non-core
Sichuan, Garzê, Batang	F01, F02	H63, H64	F01H63, F02H64	2	Unique	non-core
Sichuan, Garzê, Dawu	F06	H20	F06H20	1	Unique	non-core
Sichuan, Garzê, Dêgê	F01	H60	F01H60	1	Unique	non-core
Sichuan, Garzê, Garzê	F01	H58	F01H58	1	Unique	non-core
Sichuan, Garzê, Luhuo	F01	H57	F01H57	1	Unique	non-core
Sichuan, Garzê, Sêrtar	F01	H56	F01H56	1	Unique	non-core
Sichuan, Garzê, Sêrxü	F02	H59	F01H59	1	Unique	core
Sichuan, Garzê, Yajiang	F01	H65	F01H65	1	Unique	non-core
Xizang, Lhasa, Chengguan	F01	H11	***F01H11*** ^b^	2	Shared	non-core
Xizang, Lhasa, Damxung	F01	H33	F01H33	1	Unique	core
Xizang, Lhasa, Doilungdêqên	F01	H37	F01H37	1	Unique	non-core
Xizang, Lhasa, Lhünzhunb	F01	H34	F01H34	1	Unique	non-core
Xizang, Lhasa, Maizhokunggar	F01, F16	H12, H14	F01H14, F16H12	3	Unique	non-core
Xizang, Lhasa, Nyêmo	F01	H05	***F01H05*** ^b^	1	Shared	non-core
Xizang, Nagqu, Baqên	F01	H15, H29	***F01H15***, F01H29	2	1 Unique+1 Shared	mixed
Xizang, Nagqu, Biru	F01	H04, H13, H15, H28	***F01H04***^ab^, ***F01H13***, ***F01H15***, F01H28	5	1 Unique+3 Shared	mixed
Xizang, Nagqu, Lhari	F01	H13, H30	***F01H13***, F01H30	2	1 Unique+1 Shared	non-core
Xizang, Nagqu, Seni	F01	H04, H16, H17	***F01H04***^ab^, F01H16, ***F01H17***^b^	4	1 Unique+2 Shared	core
Xizang, Nagqu, Nyainrong	F01, F07	H04	***F01H04***^ab^, F07H04	2	1 Unique+1 Shared	core
Xizang, Nagqu, Sog	F01	H04	***F01H04*** ^ab^	1	Shared	core
Xizang, Nyingch, Bomi	F01	H04, H17, H75	***F01H04***^ab^, ***F01H17***^b^, F01H75	3	1 Unique+2 Shared	core
Xizang, Nyingch, Gongbo′gyamda	F03	H46	F03H46	1	Unique	non-core
Xizang, Nyingch, Mainling	F11, F12, F21	H09, H49, H50	F11H49, ***F12H09***, F21H50	3	2 Unique+1 Shared	non-core
Xizang, Nyingch, Langxian	F20, F26	H05, H48	F20H48, F26H05	2	Unique	non-core
Xizang, Nyingch, Bayi	F11, F12, F22, F25	H09, H27	F11H27, ***F12H09***, F22H09, F25H09	4	3 Unique+1 Shared	non-core
Xizang, Nyingch, Zayü	F01, F03	H04, H76, H51	***F01H04***^ab^, F01H76, F03H51	3	2 Unique+1 Shared	mixed
Xizang, Qamdo, Baxoi	F01	H04	***F01H04*** ^ab^	1	Shared	core
Xizang, Qamdo, Dêngqên	F01	H04, H78	***F01H04***^ab^, F01H78	2	1 Unique+1 Shared	core
Xizang, Qamdo, Gonjo	F01	H35	F01H35	1	Unique	non-core
Xizang, Qamdo, Jomda	F01	H40	F01H40	1	Unique	non-core
Xizang, Qamdo, Lhorong	F01	H36	F01H36	1	Unique	core
Xizang, Qamdo, Riwoqê	F01	H04	***F01H04*** ^ab^	2	Shared	core
Xizang, Qamdo, Zhag′yab	F01	H32	F01H32	1	Unique	core
Xizang, Qamdo, Zogang	F01	H38, H39	F01H38, F01H39	2	Unique	non-core
Xizang, Shannan, Comai	F01	H07	F01H07	1	Unique	non-core
Xizang, Shannan, Cona	F04	H07	F04H07	1	Unique	non-core
Xizang, Shannan, Gyaca	F01, F03, F04	H10	F01H10, F03H10, F04H10	3	Unique	non-core
Xizang, Shannan, Lhozhag	F04	H45	F04H45	1	Unique	non-core
Xizang, Shannan, Lhünzê	F03	H07, H77	***F03H07***^b^, F03H77	2	1 Unique+1 Shared	non-core
Xizang, Shannan, Nêdong	F04	H05	***F04H05*** ^b^	1	Shared	non-core
Xizang, Shannan, Qusum	F03	H05, H07	F03H05, ***F03H07***^b^	2	1 Unique+1 Shared	non-core
Xizang, Shannan, Zhanang	F01	H05	***F01H05*** ^b^	1	Shared	non-core
Xizang, Xigazê, Dinggyê	F04	H44	F04H44	1	Unique	non-core
Xizang, Xigazê, Gamba	F01	H05	***F01H05*** ^b^	1	Shared	non-core
Xizang, Xigazê, Gyirong	F19	H43	F19H43	1	Unique	non-core
Xizang, Xigazê, Lhazê	F04	H05	***F04H05*** ^b^	1	Shared	non-core
Xizang, Xigazê, Namling	F01	H05	***F01H05*** ^b^	1	Shared	non-core
Xizang, Xigazê, Nyalam	F01	H41	F01H41	1	Unique	non-core
Xizang, Xigazê, Tingri	F01	H42	F01H42	1	Unique	non-core
Xizang, Xigazê, Samzhubzê	F01, F09	H11, H31	***F01H11***^b^, F09H31	2	1 Unique+1 Shared	non-core
Xizang, Xigazê, Yadong	F09	H47	F09H47	1	Unique	non-core
Yunnan, Diqing, Dêqên	F24	H66	F24H66	1	Unique	non-core
Yunnan, Lijiang, Yulong	F03	H21	F03H21	1	Unique	non-core

Note: List of ITS, COI and combined haplotypes detected in 75 counties. “Unique” means the combined haplotype was only detected in one county. “Shared” (in Bold Italic) means the combined haplotype was detected in more than one county. ^a^ Combined haplotypes shared by different provinces; ^b^ Combined haplotypes shared by different Prefectures within the same Province.

For the geographical origin authentication, twenty-four ITS haplotypes discriminated 24 different production areas and 78 COI haplotypes differentiated 78 production regions. Considering that one ITS haplotype may correspond to more than one (1–52) COI haplotype and, on the other hand, one COI haplotype could also correspond to more than one (1–4) ITS haplotype, the combination of ITS and COI (combined haplotype) could discriminate more. Finally, as many as 99 production regions were separated using the combined haplotype. One combined haplotype may represent different numbers (1–10) of production regions (in the county level). For example, the most widely distributed haplotype, F01H04 (one of the core haplotypes) was observed from 10 counties, including one from Qinghai (Zadoi, Yushu) and nine from Xizang, i.e. Biru, Seni, Nyainrong and Sog in Nagqu, Bomi and Zayü in Nyingch, Baxoi, Dêngqên and Riwoqê in Qamdo, whereas most combined haplotypes (81 amongst 99) were only observed in one county (Table 1 and Suppl. material 2). In other words, samples from “core” areas where haplotype F01H04 is distributed, could not be separated from each other even using the combined barcodes. On the other hand, each county has different numbers of combined haplotypes, ranging from 1–15. A total of 48 counties out of 75 (64%) were found to possess only one combined haplotype. Maqên County in Guoluo, Qinghai, contained the largest number (15) of combined haplotypes amongst all the 75 counties (Table 1).

## ﻿Discussion

The qualities of traditional Chinese medicine from different producing regions vary due to the differences in effective medicinal components. Environmental factors such as climate, soil, biology and topography may significantly impact the growth and formation of medicinal materials, resulting in variance in quality amongst production regions. Besides, the intrinsic genetic differences amongst geographic populations may also be critical for the quality and the ‘geo-herbalism’ of medical materials. The quality of the Chinese caterpillar fungus was mainly evaluated by its present appearance, primarily the specimen’s size, colour and smell. The geographic production regions were usually supposed to be highly related to those extrinsic characteristics, causing an intentional mislabelling of the production locality during the trading process and a strong desire for the authentication of its geographical origin as a result.

Using the combination of the universal DNA barcodes for fungi (ITS) and host insects (COI), 99 combined haplotypes were recognised, which discriminated 75 investigated production counties into 99 regions. If administrative regionalisation were considered, the combined haplotypes could determine the production region to provincial level in nearly all cases with only a very few exceptions observed, i.e. Xiahe County of Gannan, Gansu shared the same haplotype F01H03 with Maqên County of Guoluo, Qinghai; Minle County of Zhangye, Gansu shared haplotype F02H01 with seven counties around Qinghai Lake (Datong, Gangca, Gonghe, Huzhu, Menyuan, Qilian, Tianjun); Zadoi County of Yushu, Qinghai shared haplotype F01H04 with the other nine Xizang counties (Baxoi, Biru, Bomi, Dêngqên, Seni, Nyainrong, Riwoqê, Sog, Zayü) (Table 1). Those exceptions are geographically close to each other or their shared haplotypes are widespread, representing the main populations of the fungus. The combined haplotypes also showed ability to discriminate production regions to the prefectural level. The confusing areas that cannot be distinguished to a prefectural level include Guoluo and Yushu in Qinghai, Prefectures of Hainan, Haixi, Haidong, Haibei and Xining around Qinghai Lake (shared the haplotype F02H01) and adjacent areas of Nagqu, Qamdo and Nyingch Prefectures (Table 1; Fig. 3).

Amongst all the 75 counties investigated in this study, 39 (52%) possessed a unique combined haplotype, which means samples from those counties could be traced; 18 (24%) counties contained both unique and shared haplotypes, those counties possibly being traced if the detected samples had the unique haplotypes, or not able to be traced if shared haplotypes were seen; the remaining 18 (24%) shared haplotypes with other counties, in other words, samples from those counties could not be accurately traced to a particular county, but to a corresponding area. It is noteworthy that all counties of Yunnan (2) and Sichuan (13) possessed unique haplotypes, the number of haplotypes ranging from 1 to 5, depending on the sample sizes (Table 1). The production region of the two provinces is characterised by its remarkable topographic of steep ridges and deep valleys of the Hengduan Mountains. This region is recognised as one of the Earth’s 34 biodiversity hotspots (Mittermeier et al. 2004) and has been reported as a diversity centre of the fungus *O.sinensis* and its host insects (Zhang et al. 2009; Quan et al. 2014a, b; Dai et al. 2019; Dai et al. 2020).

Up to now, a total of 32 ITS haplotypes have been identified from our own collections, amongst which eight ITS haplotypes (Li et al. 2021a) were not included in this study because we could not amplify COI sequences from the corresponding specimens. In contrast, Zhang et al. (2009) defined eight ITS haplotypes from 56 isolates across 10 populations. A different criterion has been used in their study which could affect the haplotype count. A more recent study by Dai et al. (2020) identified as many as 111 ITS haplotypes from 948 individuals across 96 sampled populations. The explosion of the ITS haplotype number was partly contributed by their extensive sampling size and also caused by the inclusion of numerous un-trimmed sequences from GenBank. However, some GenBank sequences may contain errors introduced during PCR or sequencing. Additionally, 18 haplotypes (Hap77–Hap90, Hap95, Hap96, Hap98 and Hap99) identified as ‘Clade VII’ in the study by Dai et al. (2020) were likely misidentified and may represent another distinct species *O.laojunshanensis* (Chen et al. 2011). Furthermore, Lu (2022) suggested that the haplotypes in ‘Clade VI’ and ‘Clade VIII’ were probably not from *O.sinensis*, but from other closely-related yet undescribed species. It is noteworthy that pseudogenic ITS sequences were not considered and included in this study. Those pseudogenes would contribute far more haplotype numbers than the functional ones according to our former studies (Li et al. 2013, 2020). ITS pseudogenes may provide valuable information in analysing species diversification and dispersal and also in authentication of geographic origin of this fungus.

In the present study, 215 individuals (specimens) from 75 counties were included, covering nearly half of the confirmed (113) and possible (55) distribution sites at the county level (Li et al. 2011). Additionally and more importantly, those specimens were all collected with detailed and credible locality information recorded and all samples were sequenced in both directions and assembled, with all ambiguous base pairs manually checked and corrected by checking the sequencing chromatograms. Sequences deposited in GenBank were not used in this study to ensure the data credibility, since single mutations could yield different haplotypes and the geographical origin authentication largely relies on the solid background database. Even though a comprehensive sampling programme was carried out in this study, collections from the remaining un-sampled regions, especially those non-core areas of Sichuan, Yunnan and south-eastern Xizang, will facilitate studies of genetic diversity, evolutionary dynamics and geographical origin authentication of this species. In addition, the sampling density to the core regions like Yushu in Qinghai, Nagqu and Qamdo in Xizang must also be improved for a better resolution. It should also be noted that the present study failed to include samples from the southern Himalayan countries of Nepal, Bhutan and India, the genetic diversity of those areas remaining largely unknown. A better picture of the core and non-core production delimiting and a more precise authentication of the geographic origin would be achieved if sampling sizes and coverage to those areas increased.

This study indicates that the host insects are more diverged than the fungus, with a total number of 78 COI haplotypes being recognised. Host insect’s diversity and complexity could be attributed to their unique life history and habitat isolation. The adult ghost moths cannot fly long distances and survive only 3–8 days (Yang et al. 1996). The complex topography resulting from the uplift of the Tibetan Plateau also limited the gene flow amongst populations (Wang et al. 2008). The host insects of *O.sinensis* showed complex vertical and regional distribution patterns on the plateau; different host species usually occupy different mountain ranges or even different sides and/or altitudes of the same mountain (Liu et al. 2005). Studying its host insects would give a better understanding of the speciation and diversification of this fungus, the co-evolution relationship between insects and their fungal associates and achieve a better resolution of defining the “core” and “non-core” production regions. Extensive insect specimens from different regions of the whole production area must be collected, identified and/or described by the taxonomists rather than just analysing sequences of one or certain more DNA fragments.

## ﻿Conclusions

The price of *O.sinensis* varies greatly amongst different production regions even with the same exterior quality, while natural products from different localities are hardly being distinguished from their appearance, resulting in confusion during trading processes. It leads to the demand of a reliable way to trace the geographical origin of this fungus. The present study developed a DNA barcoding-based method which uses ITS and COI, i.e. two universal DNA barcodes for identifying fungal and insect species, respectively. As many as 24 ITS and 78 COI haplotypes were recognised from 215 individuals that were collected from 75 different geographic localities (county level) and ninety-nine combined haplotypes were defined using both ITS and COI. The combined haplotype analysis showed an excellent performance in the geographical origin authentication of the fungus, discriminating the 75 investigated production counties into 99 distinct regions. Additionally, haplotype analysis was also found capable to define the “core” and “non-core” production regions.

## ﻿Additional information

### Author ORCIDs

Yi Li https://orcid.org/0000-0002-3482-5487

Jiao-Jiao Lu https://orcid.org/0000-0002-0916-8294

Lan Jiang https://orcid.org/0000-0002-4097-1341

Ke Wang https://orcid.org/0000-0002-5153-8498

Chao Ma https://orcid.org/0000-0002-9509-4489

Rui-Heng Yang https://orcid.org/0000-0001-5442-9388

Cai-Hong Dong https://orcid.org/0000-0002-2558-3404

Yi-Jian Yao https://orcid.org/0000-0002-7158-2963

### Data availability

All the sequences obtained in this study have been deposited in GenBank (https://www.ncbi.nlm.nih.gov/) under the accession nos. OR652460–OR652582 and OR669738–OR669952 for ITS and COI, respectively. The ITS and COI alignments have been made open-access through TreeBase and could be accessed through the link http://purl.org/phylo/treebase/phylows/study/TB2:S31885.

## Supplementary materials

Supplementary material 1 Sampling sizes, haplotype numbers and core/non-core definition for each provinces Data type xlsx https://binary.pensoft.net/file/1295715This dataset is made available under the Open Database License (http://opendatacommons.org/licenses/odbl/1.0/). The Open Database License (ODbL) is a license agreement intended to allow users to freely share, modify, and use this Dataset while maintaining this same freedom for others, provided that the original source and author(s) are credited.Yi Li, Jiao-Jiao Lu, Ya-Bin An, Lan Jiang, Hai-Jun Wu, Ke Wang, Dorji Phurbu, Jinmei Luobu, Chao Ma, Rui-Heng Yang, Cai-Hong Dong, Yi-Jian Yao

Supplementary material 2 Specimen information and haplotype classification of *Ophiocordycepssinensis* used in this study Data type xlsx https://binary.pensoft.net/file/1295716This dataset is made available under the Open Database License (http://opendatacommons.org/licenses/odbl/1.0/). The Open Database License (ODbL) is a license agreement intended to allow users to freely share, modify, and use this Dataset while maintaining this same freedom for others, provided that the original source and author(s) are credited.Yi Li, Jiao-Jiao Lu, Ya-Bin An, Lan Jiang, Hai-Jun Wu, Ke Wang, Dorji Phurbu, Jinmei Luobu, Chao Ma, Rui-Heng Yang, Cai-Hong Dong, Yi-Jian Yao
